# Peat-Based Organomineral Fertilizers Inoculated with *Bacillus* spp. Improve Lettuce Growth and Nutrient Accumulation Under Contrasting Growing Conditions

**DOI:** 10.3390/plants15132019

**Published:** 2026-06-30

**Authors:** Hamilton César de Oliveira Charlo, Sofia Isabel Almeida Pereira, Édimo Fernando Alves Moreira, Guilherme Dagrava, Arcângelo Loss, Ana Isa Marquez Rocha Machado, José Luiz Rodrigues Torres, Gislaine Fernandes

**Affiliations:** 1 Department of Agronomy, Federal Institute of the Triângulo Mineiro (IFTM), Uberaba Campus, Rua João Batista Ribeiro 4000, Uberaba 38064-790, MG, Brazil; 2 CBQF—Centro de Biotecnologia e Química Fina—Laboratório Associado, Escola Superior de Biotecnologia, Universidade Católica Portuguesa, Rua Diogo Botelho 1327, 4169-005 Porto, Portugal; 3 Department of Rural Engineering, Center for Agricultural Sciences, Federal University of Santa Catarina (UFSC), Rodovia Admar Gonzaga 1346, Itacorubi, Florianopolis 88034-001, SC, Brazil

**Keywords:** *Lactuca sativa* L., organomineral fertilizers, pelletized fertilizers, peat-based formulations, bioinoculants, *Bacillus* spp.

## Abstract

This study evaluated the effects of peat-based organomineral fertilizers with different compositions and *Bacillus* spp. inoculation on the growth and nutrient accumulation of loose-leaf lettuce grown under summer and winter conditions. Two independent greenhouse experiments were conducted using a randomized complete block design with eight treatments: no basal fertilized control (T1); conventional mineral fertilization (T2); peat-based organomineral fertilizers containing 50% (T3), 40% (T4), or 30% peat (T5); and the corresponding formulations supplemented with *Bacillus subtilis*, *Bacillus megaterium*, and *Bacillus aryabhattai* (T6–T8). All fertilized treatments were standardized to supply the same P rate. Multivariate analyses revealed a strong effect of fertilization strategy on plant growth and nutritional status. In both seasons, fertilized treatments significantly outperformed the control, while organomineral fertilizers performed similarly to or better than conventional mineral fertilization. The greatest shoot fresh mass and nutrient accumulation were observed in formulations containing lower peat proportions and higher mineral nutrient density, particularly when combined with *Bacillus* spp. inoculation. In the summer experiment, the 40% peat formulation supplemented with *Bacillus* spp. (T7) produced the highest shoot fresh mass (197.57 g plant^−1^), whereas in the winter experiment the highest value was obtained with the 30% peat formulation supplemented with *Bacillus* spp. (T8; 157.86 g plant^−1^). These treatments also exhibited greater accumulation of macronutrients and micronutrients, particularly N, P, K, Fe, Mn, and Zn. The results indicate that the performance of peat-based organomineral fertilizers was influenced by the balance between the organic matrix and mineral fraction, as well as by seasonal growing conditions. In addition, *Bacillus* spp. inoculation was associated with improved performance of formulations with greater mineral nutrient density but did not compensate for less favorable fertilizer compositions. Under the conditions evaluated, peat-based organomineral fertilizers containing lower peat proportions and supplemented with *Bacillus* spp. performed similarly to or better than conventional mineral fertilization and promoted greater lettuce growth and nutrient accumulation than the non-fertilized control. These findings are limited to a single lettuce cultivar grown in pots under greenhouse conditions across two seasonal experiments conducted at one location.

## 1. Introduction

The intensification of vegetable production has increased the demand for fertilization strategies capable of improving nutrient availability while sustaining crop productivity. In leafy vegetables such as lettuce (*Lactuca sativa* L.), which are characterized by rapid growth and a short production cycle, crop performance depends strongly on the timely availability of nutrients and the synchronization between nutrient supply and plant demand [1]. Although conventional mineral fertilization remains the predominant practice in commercial production systems, its efficiency may be limited by nutrient losses and low nutrient recovery, particularly under intensive cultivation conditions [2,3].

Organomineral fertilizers have emerged as an alternative strategy that combines mineral nutrient sources with organic matrices, integrating the immediate nutrient availability provided by mineral fertilizers with the beneficial physical and chemical properties associated with organic materials. The organic fraction has been reported to contribute to nutrient retention and improvements in soil properties, whereas the mineral fraction supplies nutrients in forms readily available for plant uptake [4,5]. However, the agronomic performance of organomineral fertilizers depends largely on the balance between their organic and mineral components, which directly influences nutrient availability throughout the crop cycle [6,7].

Beyond fertilizer composition, formulation technologies play an important role in product handling and field application. Pelletization is widely used in organomineral fertilizer production because it improves physical uniformity, reduces dust generation, enhances flowability, and facilitates application using conventional equipment [8,9]. Pelletized formulations also enable the co-delivery of organic, mineral, and biological components within a single product, providing a practical platform for integrated nutrient management.

The integration of conventional fertilization strategies with plant growth-promoting rhizobacteria (PGPR), particularly species of the genus *Bacillus*, has attracted increasing attention as a means of improving nutrient acquisition and enhancing crop growth and productivity. Among the strains used in the present study, *Bacillus megaterium* CNPMS B119 and *Bacillus subtilis* CNPMS B2084 have been reported to increase P acquisition and maize grain yield under field conditions in Brazil. These strains exhibit several plant growth-promoting traits associated with P dynamics, including phosphate solubilization and mineralization, as well as production of indole-3-acetic acid-like compounds, siderophores, exopolysaccharides, biofilms, and phosphatases [10]. Similarly, *Bacillus aryabhattai* CMAA 1363 has been shown to promote plant growth and increase crop productivity in maize and forage grasses [11,12]. More recently, Barbosa et al. [13] reported that this strain enhanced maize growth and improved plant tolerance to drought stress under greenhouse conditions. Together, these studies highlight the agronomic potential of the *Bacillus* strains evaluated in the present work and support their investigation within integrated organomineral fertilizer systems.

The successful adoption of microbial inoculants, however, depends not only on their biological effectiveness but also on the availability of efficient delivery systems. In large-scale grain crops such as maize and soybean, inoculants can be readily applied through mechanized planting operations. In contrast, vegetable production systems frequently rely on manual transplanting, which can limit the practicality of separate inoculant applications. Incorporating beneficial microorganisms directly into pelletized organomineral fertilizers may therefore offer an effective strategy for the simultaneous delivery of nutrients and microbial inoculants during crop establishment. Peat-based matrices are widely used as carriers for microbial products because of their favorable physical and chemical characteristics. Nevertheless, information remains scarce regarding the performance of peat-based pelletized organomineral fertilizers supplemented with microbial inoculants, particularly in lettuce production systems.

Peat-based pelletized organomineral fertilizers supplemented with *Bacillus* spp. have emerged as a potentially useful approach for integrating organic, mineral, and biological components within a single agricultural input. Although organomineral fertilizers and microbial inoculants have been extensively investigated as individual technologies, little is known about how peat proportion influences the performance of pelletized organomineral fertilizers or whether *Bacillus* inoculation modifies crop responses under contrasting growing conditions. In addition, few studies have examined the combined effects of fertilizer composition and microbial inoculation on lettuce growth and nutrient accumulation using an integrated multivariate framework.

In this context, the present study evaluated the effects of different formulations of a peat-based pelletized organomineral fertilizer supplemented with *Bacillus* spp. and described in a recently filed patent application on the growth and nutrient accumulation of loose-leaf lettuce cultivated under contrasting summer and winter conditions. We hypothesized that incorporating *Bacillus* spp. into peat-based organomineral fertilizers would enhance lettuce growth, biomass production, and nutrient accumulation relative to non-inoculated formulations of equivalent composition. We further hypothesized that the magnitude of these responses would depend on the peat-to-mineral fertilizer ratio and on seasonal environmental conditions.

## 2. Materials and Methods

Two independent experiments were conducted with loose-leaf lettuce under contrasting summer (5 January to 19 February 2024) and winter (1 July to 18 August 2024) growing conditions in Brazil. Both trials were carried out under protected cultivation in an arch-type greenhouse covered with a 150 µm light-diffusing polyethylene film and enclosed on the sides with 50% shade netting. The greenhouse was located in Uberaba, Minas Gerais, Brazil (19°45′26″ S, 47°55′27″ W; 800 m altitude).

According to the updated Köppen climate classification proposed by Beck et al. [14], the regional climate is classified as Aw, characterized by hot, rainy summers and dry winters. The region has a mean annual temperature of 22.6 °C and an average relative humidity of 68% [15]. Environmental variables inside the greenhouse, including air temperature, relative humidity, vapor pressure deficit, and photosynthetically active radiation, were not continuously monitored during the experiments. Nevertheless, both trials were conducted in the same greenhouse and managed using identical cultivation practices, differing only in the season in which they were carried out.

In both experiments, loose-leaf lettuce (*Lactuca sativa* L. cv. Isabela) was used. This cultivar was selected because it is widely grown in Brazil and is recognized for its broad adaptation to different environmental conditions, making it suitable for year-round production. Seedlings were produced in 200-cell polyethylene trays filled with a commercial substrate and managed under fertigation. Transplanting was performed 26 and 28 days after sowing in the summer and winter experiments, respectively, when seedlings had reached the four-leaf stage and exhibited a high degree of developmental uniformity, ensuring consistent establishment across treatments.

Each experimental unit consisted of a 5 dm^3^ pot filled with a dystrophic Red Latosol (Oxisol) with a sandy clay texture. Soil was collected from the 0–20 cm layer of a soybean–maize production area in sufficient quantity to conduct both experiments. Soil chemical analysis indicated a pH (CaCl_2_) of 5.7. The concentrations of Ca, Mg, and Al were 14.0, 6.1, and 0.1 mmolc dm^−3^, respectively, while potential acidity (H + Al) was 24 mmolc dm^−3^. The cation exchange capacity (CEC) at pH 7.0 was 45 mmolc dm^−3^, with a base saturation (V) of 46.63%. Available P and K contents were 8.8 and 34 mg dm^−3^, respectively, resulting in a sum of bases (SB) of 21 mmolc dm^−3^. Total organic carbon content was 0.78 g dm^−3^. Micronutrient concentrations were 0.04 mg dm^−3^ B, 0.8 mg dm^−3^ Cu, 97.8 mg dm^−3^ Fe, 11.1 mg dm^−3^ Mn, and 1.2 mg dm^−3^ Zn. The Ca/Mg, Ca/K, and Mg/K ratios were 2.3, 16.1, and 7.02, respectively.

Based on the soil analysis, liming was performed to increase base saturation to 80%, following technical recommendations for lettuce cultivation. Limestone was incorporated using a concrete mixer, after which the soil was incubated for 40 days with moisture maintained through periodic irrigation.

Both experiments followed a randomized complete block design consisting of eight treatments. Initially, fifteen plants per treatment were established individually in 5 dm^3^ pots, with each pot containing a single plant and representing an independent experimental unit. Pots were randomly allocated within blocks at the beginning of the experiment and remained in the same position throughout the cultivation period.

At harvest, all plants were evaluated individually. Before statistical analysis, measurements from consecutive predefined pairs of plants within each treatment (plants 1–2, 3–4, 5–6, and so forth) were averaged to generate composite analytical replicates. This pairing strategy was defined before data analysis and applied uniformly across all treatments. One remaining plant per treatment was excluded to maintain a balanced dataset. Statistical analyses were subsequently performed using the resulting seven composite analytical replicates per treatment. Consequently, the effective sample size used in all statistical analyses was seven replicates per treatment.

Consequently, the effective sample size used in all statistical analyses was seven replicates per treatment. The treatments were as follows: T1, topdressing-only control (no basal fertilization); T2, conventional mineral fertilization with monoammonium phosphate (MAP) and potassium chloride (KCl); T3, organomineral fertilizer containing 50% peat, 40% MAP, and 10% KCl; T4, organomineral fertilizer containing 40% peat, 50% MAP, and 10% KCl; T5, organomineral fertilizer containing 30% peat, 55% MAP, and 15% KCl; T6, the same formulation as T3 supplemented with *Bacillus* spp.; T7, the same formulation as T4 supplemented with *Bacillus* spp.; and T8, the same formulation as T5 supplemented with *Bacillus* spp. Thus, T3/T6, T4/T7, and T5/T8 represented paired formulations with identical chemical composition, differing only in the presence or absence of microbial inoculation.

The organomineral fertilizers used in treatments T3–T8 were produced according to the technology described in the patent application entitled “Peat-Based Organomineral Fertilizer, Pelletized and Supplemented with Microorganisms for Agricultural Application and Method of Production”, filed with the Brazilian National Institute of Industrial Property under application number BR 10 2026 006753 9 and developed by the first and last authors of this manuscript.

**Figure 1:**
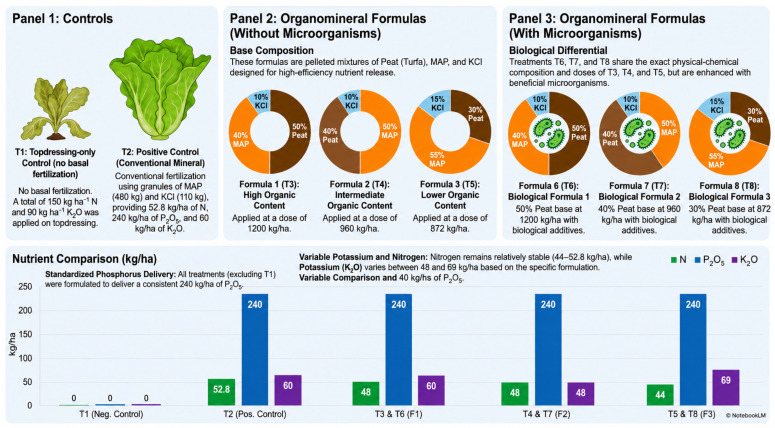
Schematic representation of the experimental treatments, including the topdressing-only control (T1; no basal fertilization), conventional mineral fertilization (T2; MAP + KCl), and peat-based organomineral fertilizers without microbial inoculation (T3–T5) or supplemented with *Bacillus* spp. (T6–T8). Organomineral formulations contained different proportions of peat, MAP, and KCl and were applied at rates of 1200, 960, and 872 kg ha^−1^, respectively. All fertilized treatments (T2–T8) were standardized to supply 240 kg ha^−1^ of P_2_O_5_. Supplementary applications of urea and KCl were incorporated into the planting furrow whenever necessary to standardize basal nutrient inputs among all fertilized treatments (T2–T8), resulting in equivalent rates of 52.8 kg ha^−1^ N, 240 kg ha^−1^ P_2_O_5_, and 69 kg ha^−1^ K_2_O.

Briefly, the formulations were produced by combining standardized Brazilian peat as a humic organic matrix, at proportions of 30, 40, or 50% of the formulation, with MAP and KCl as mineral nutrient sources in proportions adjusted to meet crop nutritional requirements (Figure 1). In treatments T6, T7, and T8, *Bacillus subtilis* CNPMS B2084, *Bacillus megaterium* CNPMS B119, and *Bacillus aryabhattai* CMAA 1363 were incorporated in spore form during fertilizer production. The fertilizers were applied 30 days after pelletization, at which time microbial viability was 2.0 × 10^6^ CFU g^−1^ of product, indicating that viable bacterial cells were retained following pelletization and drying. Shelf life evaluations demonstrated that the formulations maintained viable bacterial populations of at least 2.0 × 10^5^ CFU g^−1^ after 12 months of storage.

Fertilizer production involved preparation of the microbial inoculum, incorporation into the peat-based organomineral matrix, moisture adjustment, pelletization, and controlled drying. The resulting cylindrical pellets were approximately 6 mm in length and 3 mm in diameter and exhibited adequate physical stability for handling and application. The system was designed to integrate mineral nutrient supply, organic matter input, and microbial inoculant delivery within a single fertilizer product. Additional details regarding the production process are available in patent application BR 10 2026 006753 9 [16].

Fertilizer treatments were applied as basal fertilization 2–4 h before transplanting in an open furrow positioned in the center of each pot according to the rates established for each treatment (Figure 1). For fertilized treatments, a reference rate of 240 kg ha^−1^ of P_2_O_5_ was adopted. To ensure equivalent nutrient supply among treatments, supplementary applications of urea and KCl were incorporated into the planting furrow whenever necessary. Consequently, all fertilized treatments (T2–T8) received identical basal nutrient rates corresponding to 52.8 kg ha^−1^ N, 240 kg ha^−1^ P_2_O_5_, and 69 kg ha^−1^ K_2_O. Thus, treatment differences were attributable to fertilizer composition and the relative proportions of peat and mineral components rather than to differences in total nutrient supply.

Treatment T1 received no basal fertilization; however, it received the same topdressing fertilization as all other treatments. Topdressing fertilization was standardized across treatments because the objective of the study was to evaluate the effects of different basal fertilization strategies. This approach minimized potential confounding effects associated with differences in N and K availability during crop development. A total of 150 kg ha^−1^ N and 90 kg ha^−1^ K_2_O was applied throughout the crop cycle using urea (45% N) and KCl (56% K_2_O), respectively. To reduce the risk of salt injury and improve nutrient distribution, the total fertilizer rate was divided into three equal applications performed at 10, 20, and 30 days after transplanting, following local crop recommendations.

Irrigation was applied daily using an automated drip irrigation system equipped with two emitters per pot. Within each experiment, all pots received the same irrigation volume regardless of treatment. Irrigation volumes were adjusted according to plant age and season using the minimum drainage criterion, whereby irrigation was increased until the pots exhibiting the greatest plant development showed slight drainage after water application. This strategy was adopted to maintain soil moisture close to field capacity while minimizing excessive drainage losses and preventing water limitation throughout the crop cycle. Accordingly, irrigation was standardized across all treatments to ensure uniform water supply, although differences in plant growth could have resulted in different water demands among treatments.

In the summer experiment, irrigation volumes were 130, 150, 170, 210, 260, 330, and 400 mL pot^−1^ day^−1^ during the periods 0–10, 11–20, 21–25, 26–30, 31–35, 36–40, and 41–45 days after transplanting, respectively. In the winter experiment, irrigation volumes were 100, 120, 140, 160, 190, 230, 260, and 290 mL pot^−1^ day^−1^ during the periods 0–10, 11–20, 21–25, 26–30, 31–35, 36–40, 41–45, and 46–48 days after transplanting, respectively.

Plants reached commercial harvest at 45 and 48 days after transplanting (DAT) in the summer and winter experiments, respectively. At harvest, plants were cut at the soil surface and evaluated for head height (HH), head diameter (HD), and head fresh mass (HFM). Head height and diameter were measured using a graduated ruler, whereas fresh mass was determined using a digital scale.

The harvested heads were subsequently prepared for nutrient analysis by washing with water and neutral detergent, followed by rinsing with running water and a final rinse with distilled and deionized water. Plant material was placed in paper bags and dried in a forced-air oven at 65 °C for 72 h until constant mass. Head dry mass (HDM) was then determined, and the dried material was ground for determination of N, P, K, Ca, Mg, S, B, Cu, Fe, Mn, and Zn concentrations according to the methodology described by EMBRAPA [17]. Nutrient accumulation per plant was subsequently calculated.

Data from each experiment were analyzed and presented independently because the study was designed to evaluate fertilizer responses under two contrasting seasonal growing conditions rather than to formally test the effect of season itself. Since the experiments were conducted during different growing periods under distinct environmental conditions, each experiment was treated as an independent dataset.

The data were subjected to multivariate analysis of variance (MANOVA) because multiple response variables were evaluated simultaneously (*n* = 15). Wilks’ Lambda was used as the test statistic owing to its robustness to moderate violations of multivariate normality and homogeneity assumptions [18].

No missing values occurred in the summer experiment. In the winter experiment, three experimental units were lost, corresponding to 3 out of 54 experimental units (5.56%). Given the low proportion of missing data, multivariate analyses were conducted using complete cases only, thereby avoiding the introduction of additional assumptions associated with data imputation.

When MANOVA indicated significant treatment effects, canonical discriminant analysis (CDA) was performed to evaluate multivariate separation among treatments and identify the variables contributing most strongly to treatment discrimination. An exploratory analysis combining principal component analysis (PCA) and hierarchical cluster analysis using the UPGMA algorithm and Euclidean distance as the dissimilarity measure was also conducted to facilitate visualization of treatment grouping patterns. Finally, boxplots were generated to visualize the distribution of biometric and nutritional variables. All analyses were performed using R software version 4.5.1 (R Foundation for Statistical Computing, Vienna, Austria) [19], adopting a significance level of 5% when appropriate.

## 3. Results

### 3.1. Multivariate Analysis of Agronomic Traits and Nutrient Accumulation in Loose-Leaf Lettuce Under Summer Conditions

**Figure 2:**
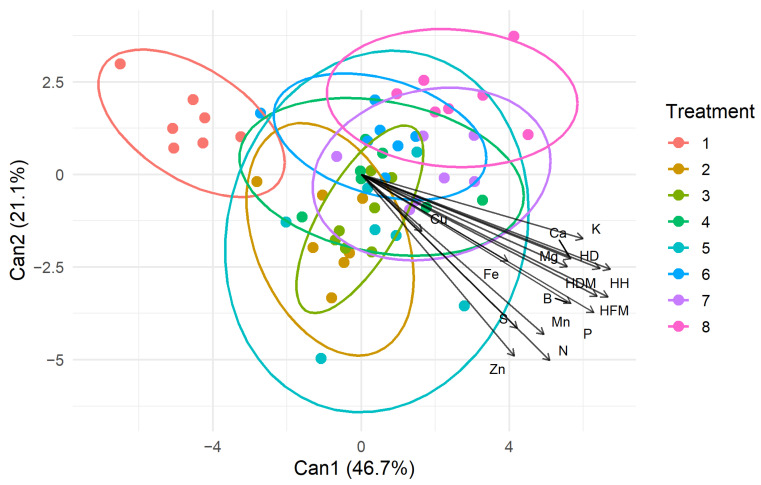
Canonical discriminant analysis (CDA) of agronomic traits and nutrient accumulation in loose-leaf lettuce grown under summer conditions and subjected to different fertilization strategies. Treatments consisted of T1, topdressing-only control (no basal fertilization); T2, conventional mineral fertilization; T3–T5, peat-based organomineral fertilizers; and T6–T8, the corresponding formulations supplemented with *Bacillus* spp. HFM, head fresh mass; HDM, head dry mass; HD, head diameter; HH, head height; N, nitrogen; P, phosphorus; K, potassium; Ca, calcium; Mg, magnesium; S, sulfur; B, boron; Cu, copper; Fe, iron; Mn, manganese; and Zn, zinc.

The multivariate analysis of variance revealed significant treatment effects on the combined set of growth and nutrient accumulation variables (Wilks’ Lambda = 0.0031, F(105, 188.53) = 2.63, *p* < 0.001). Canonical discriminant analysis (CDA) showed clear separation among treatments, with the first two canonical axes accounting for 67.8% of the total variation (Can1 = 46.7% and Can2 = 21.1%; Figure 2). The topdressing-only control (T1) occupied a distinct position in the canonical space, indicating a markedly different agronomic and nutritional profile relative to the fertilized treatments.

The first canonical axis was primarily associated with biomass production and nutrient accumulation variables. Canonical structure coefficients indicated that HFM, HDM, HH, HD, P, and K accumulation were the traits most strongly correlated with Can1 and therefore contributed most to treatment discrimination. Fertilized treatments were generally positioned along the positive portion of Can1, which was associated with greater plant growth and nutrient accumulation.

**Figure 3:**
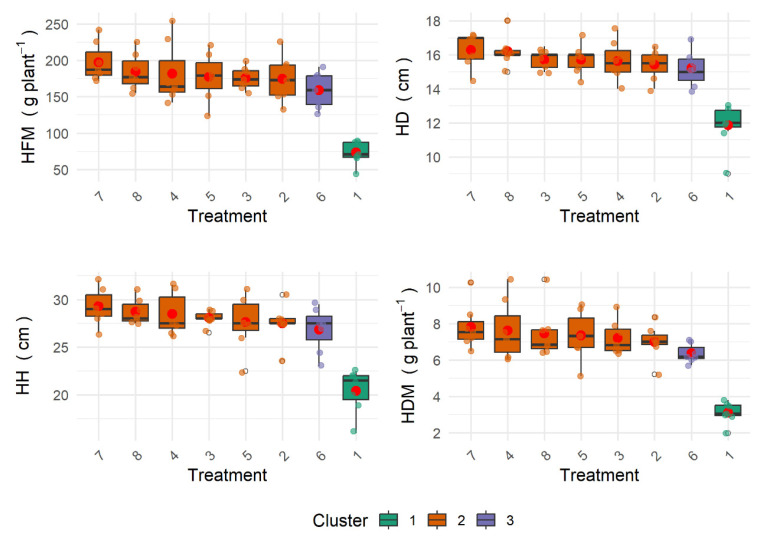
Distribution of agronomic traits in loose-leaf lettuce grown under summer conditions under different fertilization strategies. Treatments were grouped according to the multivariate similarity patterns identified by hierarchical cluster analysis. Boxes represent the interquartile range (IQR), the central line indicates the median, whiskers represent data dispersion beyond the quartiles, colored points correspond to individual observations, and red dots indicate treatment means. Treatments consisted of T1, topdressing-only control (no basal fertilization); T2, conventional mineral fertilization; T3–T5, peat-based organomineral fertilizers; and T6–T8, the corresponding formulations supplemented with *Bacillus* spp. HFM, head fresh mass; HDM, head dry mass; HD, head diameter; and HH, head height.

The distributions of agronomic variables further illustrated these differences (Figure 3). All fertilized treatments substantially outperformed T1, highlighting the importance of basal fertilization for lettuce growth under the conditions of this study. Among the fertilized treatments, T7 showed the highest agronomic performance, reaching 197.57 g plant^−1^ of shoot fresh mass and 7.82 g plant^−1^ of shoot dry mass. Treatments T4, T5, T8, and conventional mineral fertilization (T2) also exhibited high biomass production, with shoot fresh mass values ranging from 174.86 to 184.57 g plant^−1^. In contrast, T1 produced only 73.43 g plant^−1^ of shoot fresh mass and 3.10 g plant^−1^ of shoot dry mass.

**Figure 4:**
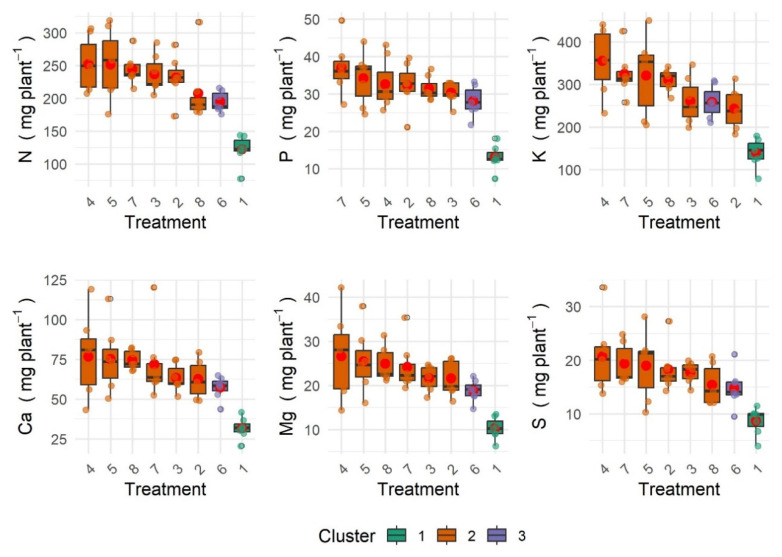
Distribution of macronutrient accumulation in loose-leaf lettuce grown under summer conditions under different fertilization strategies. Treatments were grouped according to the multivariate similarity patterns identified by hierarchical cluster analysis. Boxes represent the interquartile range (IQR), the central line indicates the median, whiskers represent data dispersion beyond the quartiles, colored points correspond to individual observations, and red dots indicate treatment means. Treatments consisted of T1, topdressing-only control (no basal fertilization); T2, conventional mineral fertilization; T3–T5, peat-based organomineral fertilizers; and T6–T8, the corresponding formulations supplemented with *Bacillus* spp. N, nitrogen; P, phosphorus; K, potassium; Ca, calcium; Mg, magnesium; and S, sulfur.

**Figure 5:**
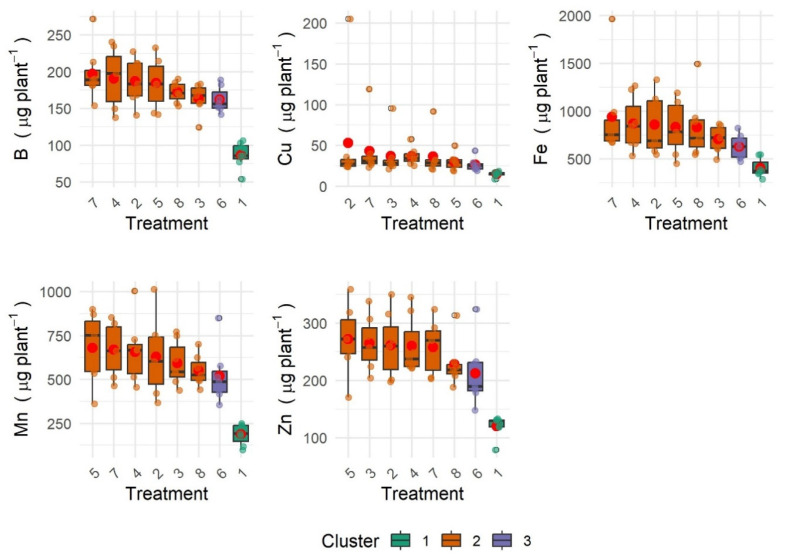
Distribution of micronutrient accumulation in loose-leaf lettuce grown under summer conditions under different fertilization strategies. Treatments were grouped according to the multivariate similarity patterns identified by hierarchical cluster analysis. Boxes represent the interquartile range (IQR), the central line indicates the median, whiskers represent data dispersion beyond the quartiles, colored points correspond to individual observations, and red dots indicate treatment means. Treatments consisted of T1, topdressing-only control (no basal fertilization); T2, conventional mineral fertilization; T3–T5, peat-based organomineral fertilizers; and T6–T8, the corresponding formulations supplemented with *Bacillus* spp. B, boron; Cu, copper; Fe, iron; Mn, manganese; and Zn, zinc.

A similar pattern was observed for nutrient accumulation (Figure 4 and Figure 5). Fertilized treatments consistently showed greater accumulation of macro- and micronutrients than T1. Treatments T7, T4, T5, T8, and T2 were generally associated with higher accumulation of N, P, K, Fe, Mn, and Zn, whereas the lowest values were consistently observed in the topdressing-only control.

**Figure 6:**
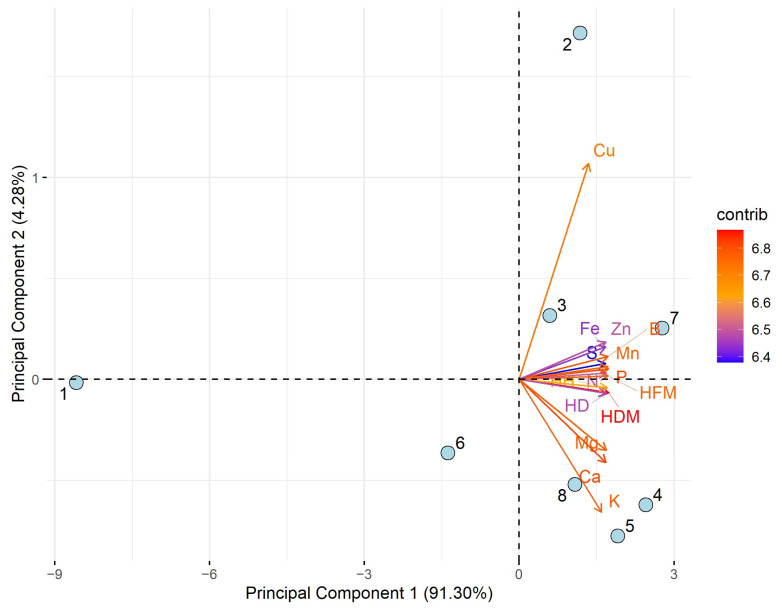
Principal component analysis (PCA) of agronomic traits and nutrient accumulation in loose-leaf lettuce grown under summer conditions under different fertilization strategies. Treatments consisted of T1, topdressing-only control (no basal fertilization); T2, conventional mineral fertilization; T3–T5, peat-based organomineral fertilizers; and T6–T8, the corresponding formulations supplemented with *Bacillus* spp. HFM, head fresh mass; HDM, head dry mass; HD, head diameter; HH, head height; N, nitrogen; P, phosphorus; K, potassium; Ca, calcium; Mg, magnesium; S, sulfur; B, boron; Cu, copper; Fe, iron; Mn, manganese; and Zn, zinc.

**Figure 7:**
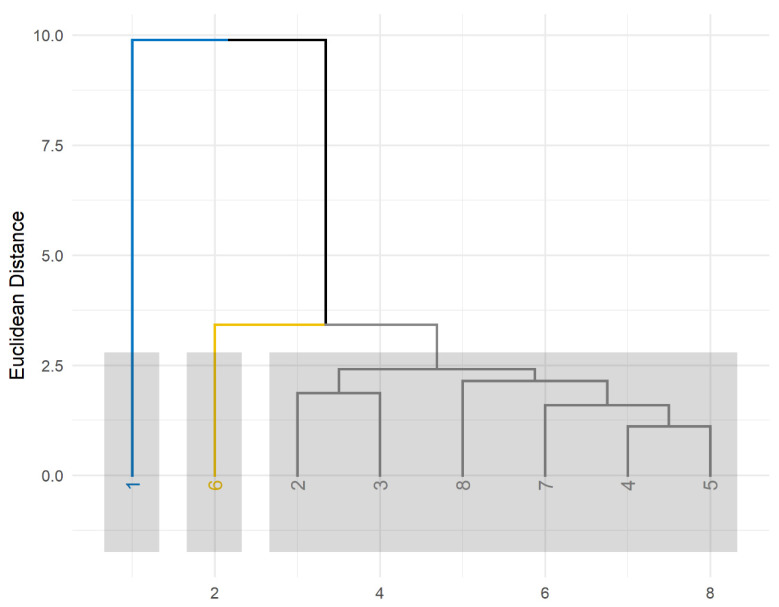
Hierarchical cluster analysis of fertilization treatments applied to loose-leaf lettuce grown under summer conditions. Treatments consisted of T1, topdressing-only control (no basal fertilization); T2, conventional mineral fertilization; T3–T5, peat-based organomineral fertilizers; and T6–T8, the corresponding formulations supplemented with *Bacillus* spp. The analysis was based on agronomic traits and nutrient accumulation data. Branch colors indicate the clusters identified by the hierarchical cluster analysis. Numbers (1–8) correspond to the experimental treatments, and the shaded rectangles indicate the clusters defined by the selected cutting level.

Principal component analysis (PCA) and hierarchical clustering provided complementary visualization of treatment relationships (Figure 6 and Figure 7). The first principal component explained 91.3% of the total variation, indicating that most treatment differences were associated with a common gradient of plant growth and nutrient accumulation. Both analyses clearly separated T1 from the fertilized treatments and highlighted the close association among T4, T5, T7, T8, and T2, reflecting their broadly similar agronomic and nutritional performance.

The Pearson correlation matrix revealed strong positive associations among biomass-related variables and most nutrient accumulation traits (Figure S1). Nutrients such as N, P, K, Fe, Mn, and Zn were positively associated with plant growth variables, indicating that greater nutrient accumulation was generally accompanied by greater biomass production. Copper showed comparatively weaker associations with treatment discrimination.

Overall, fertilized treatments consistently promoted greater growth and nutrient accumulation than the topdressing-only control. Several organomineral formulations, particularly T7, T4, T5, and T8, exhibited agronomic and nutritional performance comparable to, and in some cases numerically exceeding, that of conventional mineral fertilization (T2).

### 3.2. Multivariate Analysis and Distribution of Agronomic Traits and Nutrient Accumulation in Loose-Leaf Lettuce Under Winter Conditions

**Figure 8:**
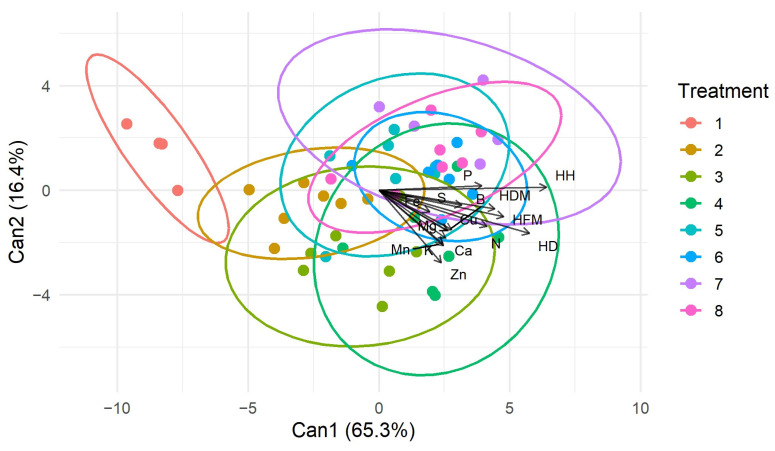
Canonical discriminant analysis (CDA) of agronomic traits and nutrient accumulation in loose-leaf lettuce grown under winter conditions under different fertilization strategies. Treatments consisted of T1, topdressing-only control (no basal fertilization); T2, conventional mineral fertilization; T3–T5, peat-based organomineral fertilizers; and T6–T8, the corresponding formulations supplemented with *Bacillus* spp. HFM, head fresh mass; HDM, head dry mass; HD, head diameter; HH, head height; N, nitrogen; P, phosphorus; K, potassium; Ca, calcium; Mg, magnesium; S, sulfur; B, boron; Cu, copper; Fe, iron; Mn, manganese; and Zn, zinc.

The multivariate analysis of variance revealed significant treatment effects on the combined set of growth and nutrient accumulation variables (Wilks’ Lambda = 0.0011, F(105, 156.53) = 2.81, *p* < 0.001). Canonical discriminant analysis (CDA) showed pronounced separation among treatments, with the first two canonical axes accounting for 81.7% of the total canonical variation (Can1 = 65.3% and Can2 = 16.4%; Figure 8). As observed in the summer experiment, the topdressing-only control (T1) occupied a distinct position in the canonical space, indicating a markedly different agronomic and nutritional profile relative to the fertilized treatments.

The first canonical axis was primarily associated with plant growth and nutrient accumulation variables. Canonical structure coefficients indicated that HH, HD, HFM, HDM, N, and P accumulation were the variables most strongly correlated with Can1 and therefore contributed most strongly to treatment discrimination. Fertilized treatments were generally positioned along the positive portion of Can1, which was associated with greater plant growth and nutrient accumulation.

**Figure 9:**
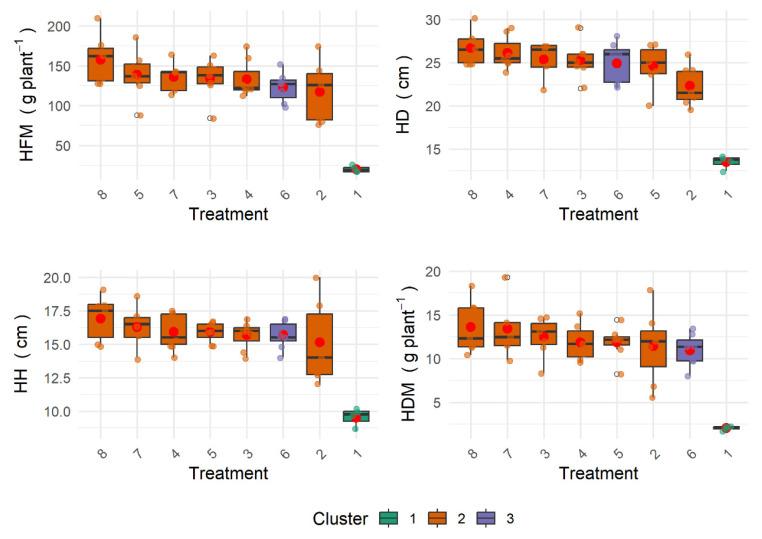
Distribution of agronomic traits in loose-leaf lettuce grown under winter conditions under different fertilization strategies. Treatments were grouped according to the multivariate similarity patterns identified by hierarchical cluster analysis. Boxes represent the interquartile range (IQR), the central line indicates the median, whiskers represent data dispersion beyond the quartiles, colored points correspond to individual observations, and red dots indicate treatment means. Treatments consisted of T1, topdressing-only control (no basal fertilization); T2, conventional mineral fertilization; T3–T5, peat-based organomineral fertilizers; and T6–T8, the corresponding formulations supplemented with *Bacillus* spp. HFM, head fresh mass; HDM, head dry mass; HD, head diameter; and HH, head height.

The distributions of agronomic variables further illustrated these differences (Figure 9). All fertilized treatments substantially outperformed T1, highlighting the importance of basal fertilization for lettuce growth under the conditions of this study. Among the fertilized treatments, T8 exhibited the highest agronomic performance, reaching 157.86 g plant^−1^ of HFM and 13.62 g plant^−1^ of HDM. Treatments T5, T7, T3, and T4 also showed high biomass production, whereas conventional mineral fertilization (T2) reached 117.14 g plant^−1^ of HFM and 11.40 g plant^−1^ of HDM. In contrast, T1 produced only 20.25 g plant^−1^ of HFM and 2.04 g plant^−1^ of HDM.

**Figure 10:**
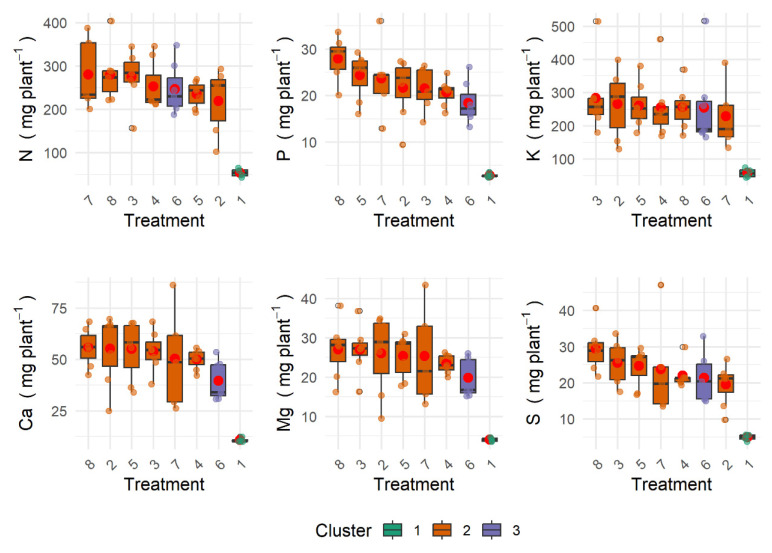
Distribution of macronutrient accumulation in loose-leaf lettuce grown under winter conditions under different fertilization strategies. Treatments were grouped according to the multivariate similarity patterns identified by hierarchical cluster analysis. Boxes represent the interquartile range (IQR), the central line indicates the median, whiskers represent data dispersion beyond the quartiles, colored points correspond to individual observations, and red dots indicate treatment means. Treatments consisted of T1, topdressing-only control (no basal fertilization); T2, conventional mineral fertilization; T3–T5, peat-based organomineral fertilizers; and T6–T8, the corresponding formulations supplemented with *Bacillus* spp. N, nitrogen; P, phosphorus; K, potassium; Ca, calcium; Mg, magnesium; and S, sulfur.

**Figure 11:**
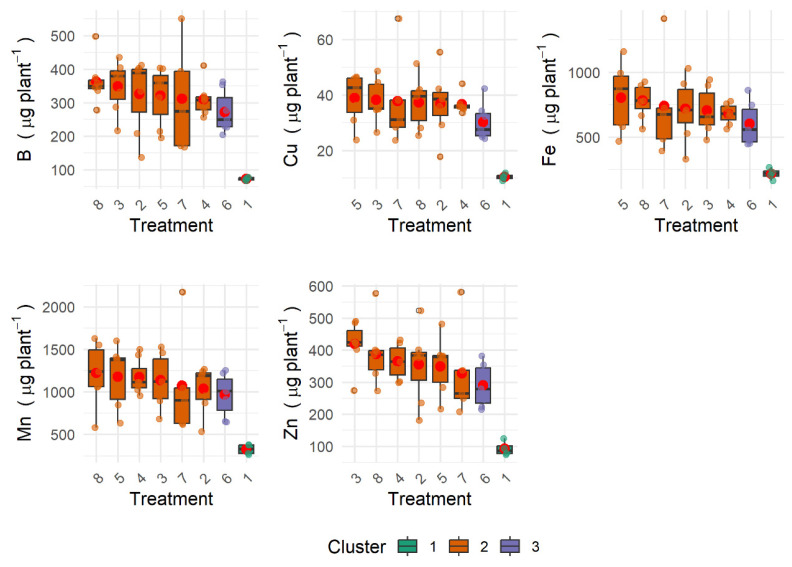
Distribution of micronutrient accumulation in loose-leaf lettuce grown under winter conditions under different fertilization strategies. Treatments were grouped according to the multivariate similarity patterns identified by hierarchical cluster analysis. Boxes represent the interquartile range (IQR), the central line indicates the median, whiskers represent data dispersion beyond the quartiles, colored points correspond to individual observations, and red dots indicate treatment means. Treatments consisted of T1, topdressing-only control (no basal fertilization); T2, conventional mineral fertilization; T3–T5, peat-based organomineral fertilizers; and T6–T8, the corresponding formulations supplemented with *Bacillus* spp. B, boron; Cu, copper; Fe, iron; Mn, manganese; and Zn, zinc.

A similar pattern was observed for nutrient accumulation (Figure 10 and Figure 11). Fertilized treatments consistently showed greater accumulation of macro- and micronutrients than T1. Treatments T8, T5, T3, T4, and T7 were generally associated with higher accumulation of N, P, K, Fe, Mn, and Zn, whereas the lowest values were consistently observed in the topdressing-only control. Conventional mineral fertilization (T2) also showed substantial nutrient accumulation and remained within the group of high-performing treatments.

**Figure 12:**
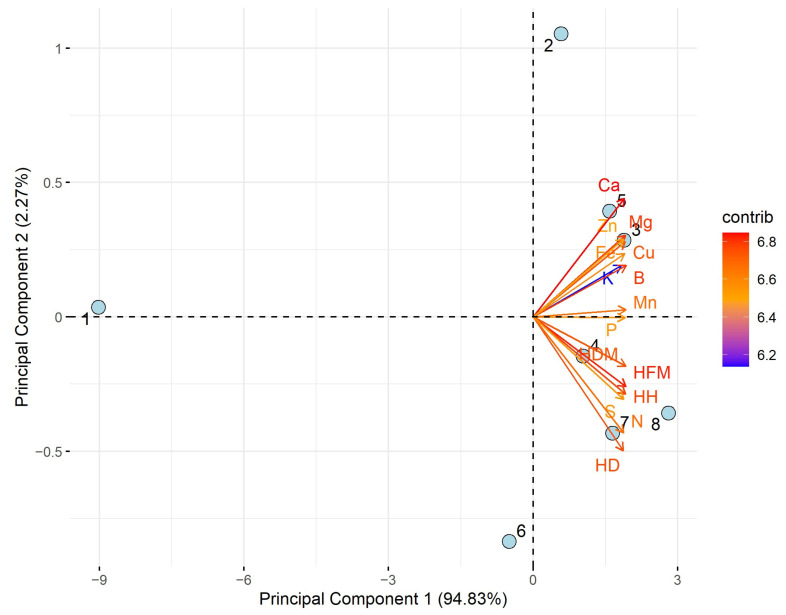
Principal component analysis (PCA) of agronomic traits and nutrient accumulation in loose-leaf lettuce grown under winter conditions under different fertilization strategies. Treatments consisted of T1, topdressing-only control (no basal fertilization); T2, conventional mineral fertilization; T3–T5, peat-based organomineral fertilizers; and T6–T8, the corresponding formulations supplemented with *Bacillus* spp. HFM, head fresh mass; HDM, head dry mass; HD, head diameter; HH, head height; N, nitrogen; P, phosphorus; K, potassium; Ca, calcium; Mg, magnesium; S, sulfur; B, boron; Cu, copper; Fe, iron; Mn, manganese; and Zn, zinc.

**Figure 13:**
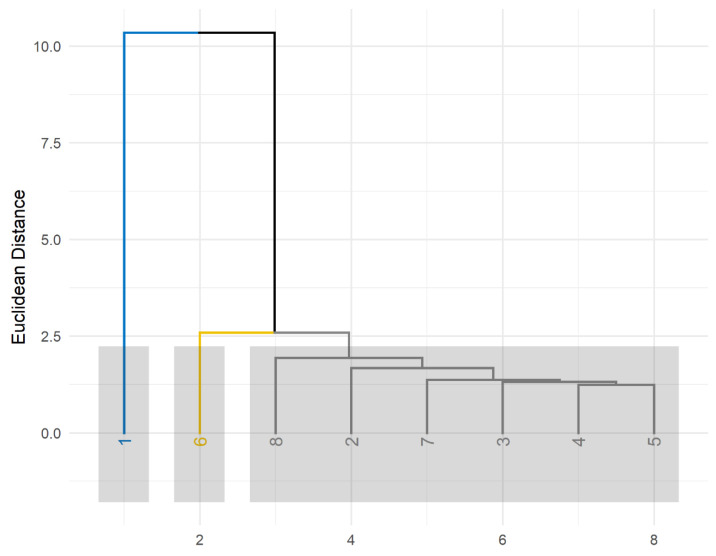
Hierarchical cluster analysis of fertilization treatments applied to loose-leaf lettuce grown under winter conditions. Treatments consisted of T1, topdressing-only control (no basal fertilization); T2, conventional mineral fertilization; T3–T5, peat-based organomineral fertilizers; and T6–T8, the corresponding formulations supplemented with *Bacillus* spp. The analysis was based on agronomic traits and nutrient accumulation data. Branch colors indicate the clusters identified by the hierarchical cluster analysis. Numbers (1–8) correspond to the experimental treatments, and the shaded rectangles indicate the clusters defined by the selected cutting level.

Principal component analysis (PCA) and hierarchical clustering provided complementary visualization of treatment relationships (Figure 12 and Figure 13). The first principal component explained 96.8% of the total variation, indicating that most treatment differences were associated with a common gradient of plant growth and nutrient accumulation. Both analyses clearly separated T1 from the fertilized treatments and highlighted the close association among T8, T5, T7, T3, T4, and T2, reflecting their broadly similar agronomic and nutritional performance.

The Pearson correlation matrix revealed strong positive associations among biomass-related variables and most nutrient accumulation traits (Figure S2). Nutrients such as N, P, K, Fe, Mn, and Zn were positively associated with plant growth variables, indicating that greater nutrient accumulation was generally accompanied by greater biomass production. As observed in the summer experiment, Cu showed comparatively weaker associations with treatment discrimination.

Overall, fertilized treatments consistently promoted greater growth and nutrient accumulation than the topdressing-only control under winter conditions. Several organomineral formulations, particularly T8, T5, T7, T3, and T4, exhibited agronomic and nutritional performance comparable to, and in some cases numerically exceeding, that of conventional mineral fertilization (T2).

## 4. Discussion

The present study demonstrated that loose-leaf lettuce performance was influenced not only by the presence of basal fertilization but also by the composition of the peat-based pelletized organomineral fertilizers. Across both experiments, all fertilized treatments consistently outperformed the topdressing-only control, underscoring the importance of adequate nutrient supply during crop establishment. However, differences among fertilized treatments indicate that the balance between the organic matrix and mineral nutrient sources was a key determinant of agronomic performance. This finding is particularly noteworthy because all fertilized treatments were standardized to receive the same P rate at planting, indicating that treatment responses were primarily associated with formulation characteristics rather than differences in total nutrient input.

A consistent response pattern was observed across both growing seasons. Formulations containing intermediate or lower peat proportions generally promoted greater biomass production and nutrient accumulation than those containing the highest proportion of peat. Although conventional mineral fertilization (T2) remained among the best-performing treatments, several organomineral formulations achieved comparable performance and, in some cases, numerically greater values for biomass production and nutrient accumulation. Collectively, these results suggest that the relative balance between peat and mineral nutrient sources played an important role in determining crop responses.

The comparatively lower performance of formulations containing 50% peat (T3 and T6) relative to those containing 40% or 30% peat suggests that increasing the proportion of the organic matrix beyond a certain threshold may not be advantageous for a fast-growing crop such as lettuce. Similar findings have been reported in studies showing that the agronomic performance of organomineral fertilizers is influenced by formulation characteristics, including the combination of organic matrices with mineral nutrient sources and their effects on nutrient availability and crop responses [5,6,7]. Nevertheless, nutrient release dynamics and soil nutrient availability were not evaluated in the present study. Therefore, although differences in nutrient availability may have contributed to the observed responses, the mechanisms underlying the superior performance of formulations containing lower peat proportions cannot be established directly from the available data.

The progression from formulations containing 50% peat (T3/T6) to those containing 40% peat (T4/T7), and subsequently 30% peat (T5/T8), was accompanied by increasing mineral nutrient density. The superior performance observed for several of these formulations suggests that the relative proportion of peat and mineral nutrient sources was an important determinant of crop performance. This interpretation is consistent with previous studies showing that the agronomic performance of organomineral fertilizers is influenced by formulation characteristics, including the relative contribution of organic matrices and mineral nutrient sources to nutrient supply and crop responses [6,7]. Taken together, these findings indicate that the performance of peat-based pelletized organomineral fertilizers in lettuce was strongly dependent on formulation. Rather than supporting a generalized advantage of higher organic matter content, the results suggest that optimizing the balance between peat and mineral nutrient sources is critical for maximizing crop performance. This provides a useful framework for interpreting the seasonal responses observed in the present study and for understanding the influence of microbial supplementation across different formulation compositions.

An important finding of the present study was the seasonal variation in the relative performance of the organomineral formulations. Although fertilized treatments consistently outperformed the topdressing-only control in both experiments, the highest-performing formulations differed between growing seasons. Treatment T7 (40% peat, 50% MAP, and 10% KCl supplemented with *Bacillus* spp.) showed the strongest agronomic response during the summer experiment, whereas T8 (30% peat, 55% MAP, and 15% KCl supplemented with *Bacillus* spp.) performed best during the winter experiment. These results suggest that the optimal balance between organic and mineral components may vary according to growing conditions.

The shift from T7 in summer to T8 in winter indicates that treatment responses were influenced not only by fertilizer composition but also by seasonal growing conditions. The contrasting performance of these formulations suggests that environmental conditions may have modified the relative effectiveness of formulations differing in peat proportion and mineral nutrient density. Because temperature and moisture strongly influence nutrient cycling and plant nutrient demand, seasonal differences in nutrient availability and crop requirements may have contributed to the distinct responses observed between experiments. This interpretation is consistent with current understanding of crop nutrition under contrasting seasonal growing conditions, although the specific environmental drivers responsible for these responses were not directly quantified in the present study.

Previous studies have shown that environmental conditions can substantially affect crop nutrient demand, nutrient availability, and fertilization efficiency [1,5]. In this context, the contrasting responses observed between seasons may reflect interactions among fertilizer composition, nutrient release patterns, and crop nutrient requirements throughout the growing cycle. Although the underlying mechanisms were not directly evaluated, the consistency of the seasonal response pattern highlights the importance of considering environmental conditions when optimizing organomineral fertilizer formulations for vegetable production.

The results also provide insight into the role of *Bacillus* spp. supplementation within pelletized organomineral fertilizers. In both experiments, some of the best-performing treatments were those supplemented with *Bacillus* spp., particularly T7 and T8. However, the response to microbial supplementation was not consistent across formulations. For example, the inoculated formulation containing 50% peat (T6) did not exhibit the same level of performance observed for T7 and T8. This pattern suggests that microbial supplementation acted primarily as an enhancer of favorable formulations rather than as a compensatory factor capable of overcoming less favorable fertilizer compositions.

The responses observed in the inoculated treatments are consistent with previous reports involving the strains used in the present study. *B. megaterium* CNPMS B119 and *B. subtilis* CNPMS B2084 have been associated with phosphate solubilization and mineralization, production of IAA-like compounds, siderophores, exopolysaccharides, biofilms, and phosphatases, resulting in increased phosphorus acquisition and maize grain yield under field conditions [10]. Similarly, *Bacillus aryabhattai* CMAA 1363 has been reported to promote plant growth, nutrient accumulation, productivity, and drought tolerance in maize and forage grasses [11,12,13,20]. Although these reports provide biological plausibility for the responses observed here, microbial persistence after soil application, root colonization, rhizosphere activity, nutrient solubilization, and root development were not assessed. Therefore, it remains uncertain whether the mechanisms previously reported for these strains were active under the conditions of the present study. Consequently, the observed responses cannot be attributed to any specific biological process and should instead be interpreted as an agronomic association between *Bacillus* supplementation and improved performance in selected formulations.

An additional aspect worth highlighting is that microbial viability was confirmed after pelletization, demonstrating that the pelletization process did not prevent short-term survival of viable *Bacillus* propagules within the fertilizer matrix. The strains were incorporated in spore form, a biological state known for its high tolerance to environmental and processing stresses, which may have contributed to their survival during pellet production.

Beyond the agronomic responses observed, the pelletized nature of the evaluated formulations represents an important technological feature of the proposed system. Pelletization facilitates handling, storage, transportation, and field application [8,9]. In the formulations evaluated here, the pelletized structure also provided a practical means of combining nutrients, organic matter, and microbial inoculants within a single product. Under the conditions evaluated, several peat-based organomineral formulations performed similarly to, and in some cases numerically exceeded, conventional mineral fertilization. Nevertheless, further studies addressing large-scale production, economic feasibility, regulatory requirements, and performance under commercial production conditions will be necessary to support broader adoption of this technology.

The experiments were conducted using a commercially relevant loose-leaf lettuce cultivar under controlled greenhouse conditions and across two contrasting growing seasons, providing a robust framework for evaluating treatment responses. Nevertheless, as with any controlled-environment study, caution is warranted when extrapolating these findings to other lettuce cultivars, leafy vegetables, soil types, and commercial production systems.

In addition, the present study focused on agronomic performance and nutrient accumulation, whereas other attributes associated with marketability and product quality, including nitrate concentration, visual quality, leaf texture, color, physiological disorders, and postharvest behavior, were not evaluated. Recent evidence indicates that greenhouse environmental conditions can substantially influence nitrate accumulation, nutritional quality, tissue integrity, and shelf life performance in leafy vegetables [21]. Therefore, future studies integrating quality and postharvest assessments would provide a more comprehensive evaluation of the commercial potential of pelletized organomineral fertilizers.

Overall, the present study demonstrated that peat-based pelletized organomineral fertilizers can sustain loose-leaf lettuce growth and nutrient accumulation at levels comparable to conventional mineral fertilization under greenhouse conditions. The results further showed that fertilizer performance was strongly influenced by formulation design, particularly the balance between peat proportion and mineral nutrient density, and that selected formulations supplemented with *Bacillus* spp. were consistently associated with high agronomic performance across contrasting growing seasons. Beyond their agronomic effectiveness, the evaluated formulations demonstrate the technical feasibility of integrating organic, mineral, and biological components within a single pelletized fertilizer product. Collectively, these findings expand current knowledge regarding peat-based organomineral fertilizers and provide a basis for future studies aimed at optimizing integrated fertilization strategies. Future research should focus on nutrient release dynamics, microbial persistence after soil application, root system responses, interactions between fertilizer composition and environmental conditions, quality and postharvest attributes, and validation across cultivars, growing environments, and commercial production systems.

## 5. Conclusions

The present study demonstrated that the performance of peat-based pelletized organomineral fertilizers in lettuce was strongly influenced by formulation characteristics. Under the conditions evaluated, all fertilized treatments promoted greater growth and nutrient accumulation than the topdressing-only control, while several organomineral formulations achieved performance comparable to conventional mineral fertilization.

Treatments T7 and T8 were among the highest-performing formulations in the summer and winter experiments, respectively, suggesting that the relative performance of fertilizer formulations may vary under contrasting growing conditions. The results further indicate that *Bacillus* spp. supplementation acted primarily as an enhancer of favorable formulations rather than as a compensatory factor for less effective fertilizer compositions.

These findings indicate that peat-based pelletized organomineral fertilizers can serve as a practical platform for integrating organic, mineral, and biological components within a single fertilizer product. Future studies should evaluate nutrient release dynamics, microbial persistence after soil application, quality and postharvest attributes, and the performance of these formulations under diverse cultivars and commercial production conditions.

## Data Availability

The datasets generated and analyzed during the study are available from the corresponding author upon reasonable request.

## Supplementary Materials

The following supporting information can be downloaded at: https://www.mdpi.com/article/10.3390/plants15132019/s1, Figure S1. Correlation matrix of agronomic traits and nutrient accumulation in loose-leaf lettuce grown under summer conditions and subjected to different treatments: T1, topdressing-only control (no basal fertilization); T2, conventional mineral fertilization; T3–T5, peat-based organomineral fertilizers; and T6–T8, the corresponding formulations supplemented with *Bacillus* spp. HFM, head fresh mass; HDM, head dry mass; HD, head diameter; HH, head height; N, nitrogen; P, phosphorus; K, potassium; Ca, calcium; Mg, magnesium; S, sulfur; B, boron; Cu, copper; Fe, iron; Mn, manganese; and Zn, zinc. Figure S2. Correlation matrix of agronomic traits and nutrient accumulation in loose-leaf lettuce grown under winter conditions and subjected to different treatments: T1, topdressing-only control (no basal fertilization); T2, conventional mineral fertilization; T3–T5, peat-based organomineral fertilizers; and T6–T8, the corresponding formulations supplemented with *Bacillus* spp. HFM, head fresh mass; HDM, head dry mass; HD, head diameter; HH, head height; N, nitrogen; P, phosphorus; K, potassium; Ca, calcium; Mg, magnesium; S, sulfur; B, boron; Cu, copper; Fe, iron; Mn, manganese; and Zn, zinc.
