# Supplementary material for: Peat-Based Organomineral Fertilizers Inoculated with Bacillus spp. Improve Lettuce Growth and Nutrient Accumulation Under Contrasting Growing Conditions
Source: Plants (Basel). 2026 Jun 30;15(13):2019. doi: 10.3390/plants15132019 (PMC13363781; doi:10.3390/plants15132019)
Supplement: Supplementary file 1 [file plants-15-02019-s001.zip › plants-4364771-supplementary.pdf]

Supplementary material

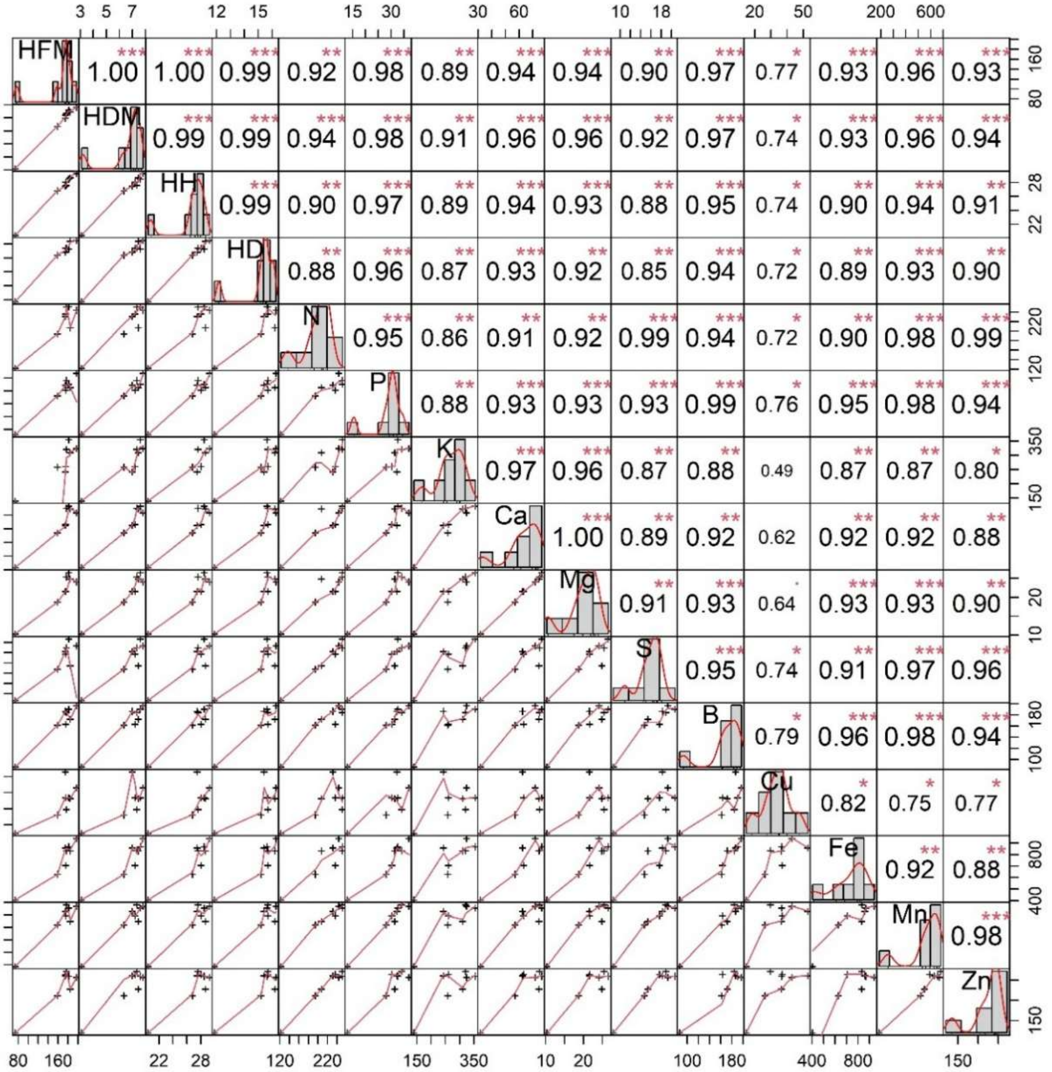

Figure S1. Correlation matrix of agronomic traits and nutrient accumulation in loose-leaf lettuce grown under summer conditions and subjected to different treatments: T1, topdressing-only control (no basal fertilization); T2, conventional mineral fertilization; T3–T5, peat-based organomineral fertilizers; and T6–T8, the corresponding formulations supplemented with *Bacillus* spp. HFM, head fresh mass; HDM, head dry mass; HD, head diameter; HH, head height; N, nitrogen; P, phosphorus; K, potassium; Ca, calcium; Mg, magnesium; S, sulfur; B, boron; Cu, copper; Fe, iron; Mn, manganese; and Zn, zinc.

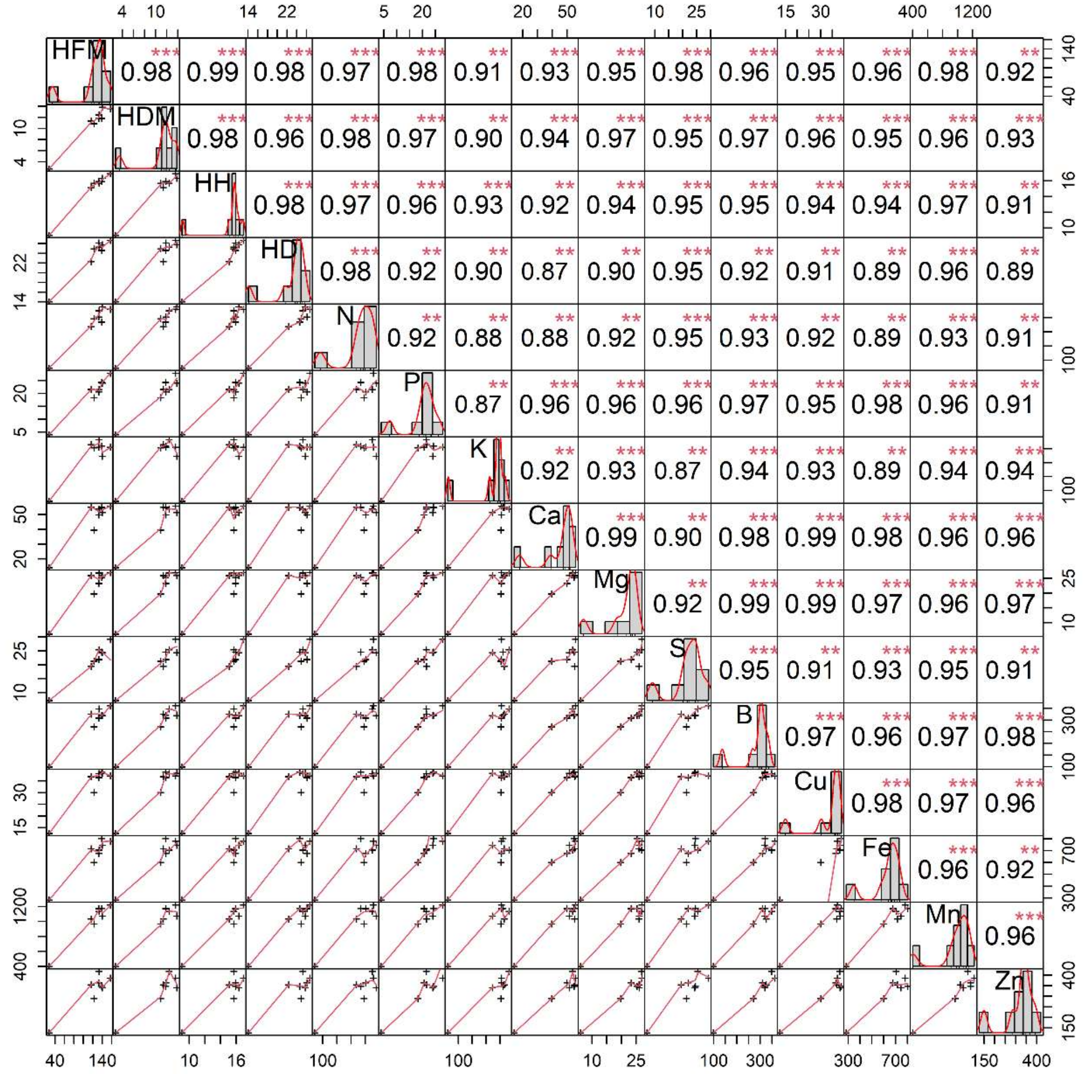

Figure S2. Correlation matrix of agronomic traits and nutrient accumulation in loose-leaf lettuce grown under winter conditions and subjected to different treatments: T1, topdressing-only control (no basal fertilization); T2, conventional mineral fertilization; T3–T5, peat-based organomineral fertilizers; and T6–T8, the corresponding formulations supplemented with *Bacillus* spp. HFM, head fresh mass; HDM, head dry mass; HD, head diameter; HH, head height; N, nitrogen; P, phosphorus; K, potassium; Ca, calcium; Mg, magnesium; S, sulfur; B, boron; Cu, copper; Fe, iron; Mn, manganese; and Zn, zinc.
